# Assessment of Interaction Behaviors of Cement-Emulsified Asphalt Based on Micro-Morphological and Macro-Rheological Approaches

**DOI:** 10.3390/ma15031070

**Published:** 2022-01-29

**Authors:** Ganghua Hu, Qing Yang, Xin Qiu, Huiqiong Liu, Yanfeng Qian, Shanglin Xiao

**Affiliations:** 1 Road and Traffic Engineering Institute, College of Engineering, Zhejiang Normal University, Jinhua 321004, China; ganghuahu@zjnu.edu.cn (G.H.); xqiu@zjnu.cn (X.Q.); huiqiongliu@zjnu.edu.cn (H.L.); yfqian@zjnu.edu.cn (Y.Q.); 2 Key Laboratory of Urban Rail Transit Intelligent Operation and Maintenance Technology & Equipment of Zhejiang Province, Zhejiang Normal University, Jinhua 321004, China

**Keywords:** cement emulsified asphalt composite binder, cement-emulsified asphalt interaction, micro-morphology, particle size distribution, macro-rheological index

## Abstract

Cement emulsified asphalt composite binder (CEACB) plays a determining role in the construction of cold recycled asphalt pavements. Understanding the interaction behaviors of cement-emulsified asphalt is very essential to promote the serviceability of CEACB. The objective of this study was to explore the interaction behaviors and mechanism of cement-emulsified asphalt associated with microstructural characteristics and to assess the interaction ability of cement-emulsified asphalt by performing macro-rheological measurements. Firstly, the physico-chemical interaction of cement-emulsified asphalt was qualitatively discussed by analyzing the difference of characteristic peaks based on Fourier transform infrared (FTIR) spectrometer. Secondly, the micro-morphological evolution behaviors of CEACB attributing to the cement-emulsified asphalt interaction were investigated by using a fluorescence microscope (FM) and laser particle size analyzer (LPSA). Thirdly, the microstructural characteristics of CEACB were studied by observing the spatial network structure through the scanning electron microscopy (SEM). Finally, the macro-rheological index based on dynamic rheological shear (DSR) test was proposed to evaluate the interaction ability of cement-emulsified asphalt. The results show that the cement-emulsified asphalt interaction is merely a physical blending process due to the occurrence of no new characteristic peaks in the FTIR spectrum except for cement hydration products. The cement-emulsified asphalt interaction in early-age CEACB could be reflected by the aggregation process among asphalt droplets and the adsorption action of cement particles to asphalt droplets. A reasonable ratio of cement to emulsified asphalt could promote the formation of the denser spatial network structure of CEACB along with cement hydration products growing and interweaving with asphalt films. The *K-B-*$G^{*}$ index based on macro-rheological properties of CEACB with full consideration of cement hydration process is very suitable for evaluating the interaction ability of cement-emulsified asphalt under the condition of different cement proportions and curing time. The research would provide the support for understanding the natural properties of CEACB and promote the improvement of the mechanical performance of cold recycled asphalt pavements.

## 1. Introduction

Cold recycling technology has great significance for promoting the waste utilization and the environmental protection due to the benefits of high Reclaimed Asphalt Pavement (RAP) disposal rate, low energy consumption and light environmental pollution, etc. [1,2,3,4,5]. With the improvement of emulsified asphalt techniques, cold recycled asphalt emulsion mixture (CRAEM) as a promising road material has been extensively utilized in the projects of pavement maintenance and rehabilitation. As far as CRAEM is concerned, recycled aggregates could be regarded as a dispersed phase evenly distributed in cement emulsified asphalt composite binder (CEACB) which contributes to the performance improvement of CRAEM through bonding recycled aggregates and filling voids [6]. However, CEACB is actually a composite material composed of cement, emulsified asphalt and other additives, which has the characteristics of integrating the rigidity of inorganic cement and the flexibility of organic emulsified asphalt. It is observed to note that the interaction behaviors between cement and emulsified asphalt (cement-emulsified asphalt) have a significant influence on the occurrence of various diseases in cold recycled asphalt pavements such as cracking, rutting, moisture damage, etc. [7,8,9]. 

Recently, the cement-emulsified asphalt interaction has been interpreted by the interrelated, interactive and restricted processes along with the hydration of cement and the demulsification of emulsified asphalt. The heat release deriving from the cement hydration reaction could accelerate the emulsified asphalt demulsification, and the formation of asphalt films originating from emulsified asphalt demulsification would delay the degree of the cement hydration reaction [10,11,12]. Thus, it could be concluded that the cement-emulsified asphalt interaction would dramatically affect cement hydration products and asphalt films to connect with each other and change the staggered support framework of CEACB. Therefore, more attention from abundant experts and scholars in road engineering industry was dedicated to the research of the interaction between cement and emulsified asphalt for improving viscoelastic characteristics and mechanical properties of CEACB [13,14,15]. 

Over the past decades, various internal and external factors such as temperature, water evaporation, cement composition, cement proportion, emulsifier type, emulsified asphalt content, etc., affecting the process and the degree of the cement hydration and the emulsified asphalt demulsification occurred in the cement emulsified asphalt materials had been widely concerned [16,17,18]. For instance, through investigating the cement hydration development, Tan et al. [19] proposed that the retarding impact was closely related to the type of emulsifier and its proportion in emulsified asphalt. Miljković et al. [20] studied the effect of a cationic emulsified asphalt and indicated that the type of emulsifier was a critical factor to improve the mechanical performance of cement emulsified asphalt mortar. Besides, the cement content and types would dramatically affect the process of the cement hydration and the emulsified asphalt demulsification compared with other influencing factors [21,22]. Wang et al. [23] investigated the cement hydration development of cement emulsified asphalt mortar, indicating that the hydration rate of cement would decrease with the increase in mass ratio of solid asphalt to cement. Kuz’Min et al. [24,25] demonstrated that the burned rock could be utilized as an active mineral additive to optimize the ultimate quantality of cement mortar due to its special chemical composition and physical properties. Thus, it is considered as an important research direction to promote the improvement of pavements performance of CEACB. Additionally, the processes of the cement hydration and the emulsified asphalt demulsification were studied through a thorough examination of external factors including curing time, curing temperature, water evaporation and so on [9,26,27,28]. For example, Du et al. [29] demonstrated that curing cement asphalt emulsion mixture with lower humidity could promote the growth of hydration products. Graziani et al. [30] utilized the Michaelis-Menten model to analyze the relation between evaporative water loss and indirect tensile strength, indicating that the high curing temperature could expedite the water evaporation and thus accelerate the curing rate of cold-recycled bituminous mixtures. Therefore, it could be believed that components of CEACB and the relevant influence factors would exert an important effect on the cement-emulsified asphalt interaction.

Besides, recent achievements focused on the microstructural properties of CEACB have made enormous contributions to understand the cement-emulsified asphalt interaction and improve the pavement performance of CEACB. Du et al. [8] investigated the interactive behaviors between cement particles or hydration products and emulsified asphalt evaporation residues through X-ray diffraction (XRD) and FTIR, demonstrating that interaction between them is a physical blending process in essence. It is observed that the variation of particle size was obvious in the early-age cement-emulsified asphalt during the adsorption process of cement particles to asphalt droplets through the measurement of laser particle size analyzer (LPSA) [31]. Wang et al. [32] utilized LPSA to measure the particle size change of emulsified asphalt under stress condition, which provided a new method to continuously evaluate the dynamic change of the emulsified asphalt stability. The relationship between the early-age reaction of cement hydration and the chemical stability of asphalt emulsion was also studied through the measurement of Zeta potential of asphalt droplets and the observation of microstructure of asphalt droplets by optical microscope [33]. To further reveal the demulsification behaviors of asphalt emulsion in the early-age CEACB, Ouyang et al. [34] investigated morphological evolution characteristics of asphalt droplets based on optical microscope. Leiben et al. [35] confirmed that the addition proportions of emulsified asphalt could obviously affect the damping performance of the cement emulsified asphalt mortar through dynamic mechanical analysis (DMA) and environmental scanning electron microscopy (ESEM). With the assistance of scanning electron microscopy (SEM) and computed tomography (CT), it was proved that the pore structures of cement-emulsified asphalt mixtures with different cement and emulsified asphalt contents had great influence on the mechanical properties of cement emulsified asphalt mixtures [36]. Consequently, measuring the micro-morphological characteristics and the dynamic development processes of CEACB is a beneficial practice for further exploring the behaviors and mechanism of cement-emulsified asphalt interaction.

In addition, many studies have indicated that the interaction behaviors of cement-emulsified asphalt could be qualitatively explored by viscoelastic properties and rheological parameters of CEACB. For instance, Garilli et al. [37] confirmed that the complex viscosity coefficient of the bitumen emulsion cement paste could be employed to reflect the retarding effect of asphalt emulsion on cement solidification with the increase in bitumen content. Ouyang et al. [38] applied the apparent viscosity parameter of cement asphalt emulsion paste to analyze the adsorption effect of different charged emulsified asphalts on cement particles and found that cationic asphalt droplets were more easily adsorbed by cement particles exhibiting the stronger interaction ability to cement particles. Tian et al. [39] adopted the elastic modulus to estimate the influence of A/C ratio on the formation of complicated microstructure of cement asphalt emulsion generated by both cement hydrates and asphalt agglomerates. Tian et al. [40] analyzed the strength development of cement hydration products during the process of asphalt emulsion demulsification by utilizing the complex modulus of CEACB. Ding et al. [14] applied the creep curves and the storage modulus mater curves to distinguish the mechanical properties of CEACB under the condition of different A/C ratios, determining it as viscoelastic solid material or viscoelastic fluid material. These studies were of great significance to give deep insight into the nature and capability of the cement-emulsified asphalt interaction. Nevertheless, the above rheological-based evaluation indexes have not been applied to quantitatively evaluate the interaction ability of cement-emulsified asphalt due to the complex of asphalt emulsion demulsification and cement hydration process. Referring to the outcome of previous studies, implementing the in-depth research as for CEACB is urgently needed.

Undoubtedly, previous efforts focused on various factors affecting the characteristics and behaviors of the cement emulsified asphalt materials have made significant contribution to qualitatively interpret the cement hydration and the emulsified asphalt demulsification of CEACB. However, little attention had been paid to explore the correlation between cement hydration and asphalt demulsification from the whole process from fresh state to strength formation to some extent of CEACB and to excavate reasonable indexes to characterize the interaction degree of the cement-emulsified asphalt. Therefore, the main purpose of this research was to systematically illuminate the behaviors and mechanism of cement-emulsified asphalt interaction and quantitatively evaluate the interaction ability of cement-emulsified asphalt from a multi-scale perspective. Specifically, this paper aimed to: 

(1) Analyze the difference of characteristic peaks based on FTIR to determine the physico-chemical interaction behavior between cement and emulsified asphalt.

(2) Discuss the micro-morphological evolution process through FM, LPSA and SEM tests to interpret the interaction mechanism of cement-emulsified asphalt from the perspective of whole process.

(3) Evaluate the interaction ability of cement-emulsified asphalt by selecting an appropriate macro-rheological index with consideration of cement hydration process based on macro-rheological properties of CEACB under the condition of different curing time and cement proportions. 

Consequently, the research would hopefully provide the support for the optimization design of cold recycled asphalt emulsion mixtures and the improvement of the mechanical performance of cold recycled asphalt pavements.

## 2. Materials and Methods

### 2.1. Materials

#### 2.1.1. Cement and Emulsified Asphalt

The slow-setting cationic emulsified asphalt with 60 percent solid content and the ordinary Portland cement with a strength grade of 42.5 Mpa (PO·42.5) were chosen as main components to prepare CEACB. The chemical composition of PO·42.5 cement was analyzed through the X-ray spectrometer (D8 Advance, Bruker AXS, Karlsruhe, Germany) as presented in Table 1. The main technical properties of emulsified asphalt were measured in accordance with ASTM specifications as presented in Table 2.

#### 2.1.2. CEACB

During the process of preparing CEACB, the two kinds of additives including organic silicon defoamer and polycarboxylate superplasticizer were added to satisfy the required properties of CEACB in addition to cement, emulsified asphalt and additional water [14]. The mixing proportion of CEACB were kept at the mass ratios (solid asphalt to cement, A/C) of 0.6, 1.0 and 1.4, respectively. The defoamer of 0.3% and superplasticizer of 2% relative to the total mass ratio were used to eliminate bubbles in CEACB and avoid the occurrence of uneven precipitation. The preparation process of CEACB was adopted as the following procedure. Next, additional water, defoamer and superplasticizer were fully blended, and cement was added and mixed at a slow speed for 30 seconds. The emulsified asphalt was then put into and blended with an agitator for 3 min at 120 rpm and continue to stir 1 min at 60 rpm to remove large bubbles. After all components were together mixed evenly, the specimens were placed in the humidity chamber at the constant temperature of 25 °C and the relative humidity of 65%.

### 2.2. Methods 

#### 2.2.1. Fourier Transform Infrared Spectroscopy

The characteristic peaks of cement powder, emulsified asphalt evaporation residue (EAER) and CEACB were analyzed by the Fourier transform infrared (FTIR) spectroscopy (Nicolet iS5, Thermo Fisher Scientific, Waltham, MA, USA). During the testing process, the projection mode and the attenuated total refraction mode were utilized to cement powder, and EAER and CEACB, respectively. The spectra were collected with a spectral resolution of 4 cm^−1^ in the wavenumber range of 4000–600 cm^−1^. The representative spectrum originated from an average of 16 spectra in FTIR measurements. To explore the physico-chemical process of cement-emulsified asphalt interaction, the features of functional groups were determined through characterizing the shape, position and width of serials of typical FTIR spectrum peaks. 

#### 2.2.2. Fluorescence Microscope 

The Fluorescence microscope (FM) apparatus (DM2700P, Leica, Wetzlar, Germany) with an ordinary optical mode was adopted to directly observe the micro-morphological variance characteristics of early-age CEACB at 0 min, 60 min and 90 min. In order to get the desired test results, each sample was diluted 50 times and was scanned with the magnification of 500. A representative result from three replicate experiments was employed to characterize the micro-morphology of early-age CEACB.

#### 2.2.3. Laser Particle Size Analyzer

The size of cement particles and asphalt droplets of early-age CEACB at 0 min, 30 min, 60 min, 90 min and 120 min were measured by the laser particle size analyzer (LPSA) (Bettersize2600E, Dandong Baxter instrument Co. Ltd., Dandong, China) with a range of 0.02–2600 μm. The dispersion medium was distilled water. The sample was put directly into the measuring pool of the instrument until the shading rate arrived at the requirement of 10–20%. The uniformity of the sample in the pool was achieved through the built-in ultrasonic dispersion technology. 

#### 2.2.4. Scanning Electron Microscopy 

The microstructural morphology of each CEACB was examined by the scanning electron microscopy (SEM) apparatus (EM30-AX Plus, COXEM, Daejeon, Korea) at curing time of 1 day and 7 days. Each CEACB was broken into small pieces with a hammer, and small pieces at least 5 mm from the surface were soaked in anhydrous ethanol to terminate hydration. The specimens were then placed in vacuum oven until dry at a temperature of 25 °C. Three kinds of CEACB samples with a dimension of 25 mm × 35 mm were plated with a thin protective palladium layer utilizing low vacuum sputtering before the SEM observation. Electrons were emitted on the surface of sample with magnifications of 500, 2000, 5000 and 10,000 at a voltage of 20 kV. Three replicates were performed at each experimental condition and a representative result was chosen for detailed description. 

#### 2.2.5. Dynamic Shear Rheometer 

The frequency sweep tests were performed by the dynamic shear rheometer (DSR) (MCR 302, Anton Paar, Graz, Austria) to obtain the complex shear modulus (G*) of CEACB with curing time of 1 day, 3 days, 7 days and 14 days and to quantitatively evaluate the cement-emulsified asphalt interaction based on rheological characteristics of CEACB. The 8 mm parallel plate with a 2 mm gap was adopted for rheological tests. The loading range of the frequency measurement was implemented from 0.01 to 10 Hz at the temperature of 25 °C under the controlling strain of 0.08%. To ensure an acceptable repeatability of the results, a representative result from two independent experiments was discussed for each experimental condition.

## 3. Results and Discussion

### 3.1. Microstructural Composition of CEACB

**Figure 1:**
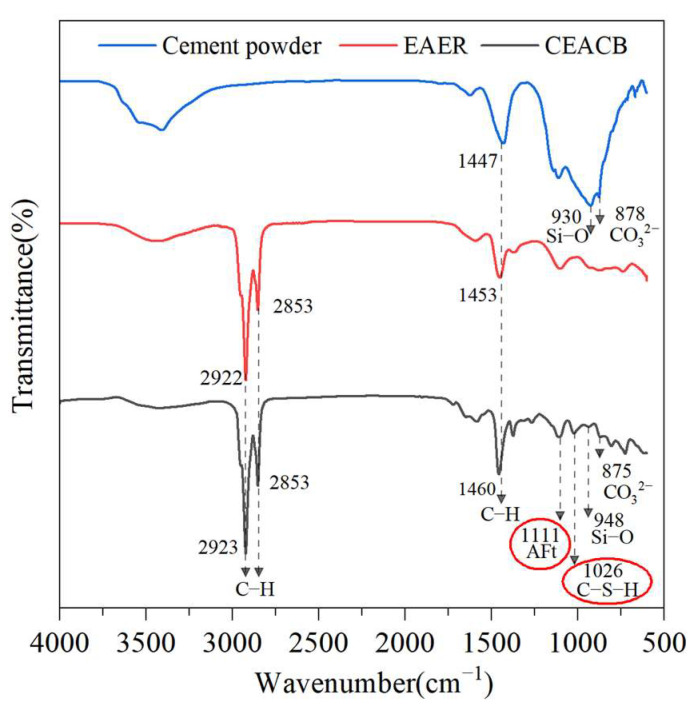
FTIR spectra of cement powder, EAER and CEACB.

The comparison of FTIR spectra among EAER, cement powder and CEACB is shown in Figure 1. The results indicate that the peaks at 2922 cm^−1^ and 2853 cm^−1^ are the main characteristic absorption peaks of EAER corresponding to the stretching vibration absorption peak of C–H bond. The peak at 1453 cm^−1^ in EAER denotes the bending vibration absorption peak of C–H bond. As for cement powder, the stretching vibration absorption peak at 930 cm^−1^ represents the Si–O bond and the absorption peak at 878 cm^−1^ is the bending vibration of the C=O double bond in CO_3_^2−^. As far as CEACB is concerned, the absorption peaks of 2922 cm^−1^ and 2853 cm^−1^ of EAER are presented in the CEACB spectrum at the same peak position. Besides, the absorption peaks of Si–O bond and C=O double bond in CO_3_^2−^ of cement powder are also observed in the similar position. It is worth mentioning that the absorption peaks at 1111 cm^−1^ and 1026 cm^−1^ in CEACB denote the ettringite (AFt) and the calcium silicate hydrate (C–S–H), respectively, which mainly derive from the new groups of SiO_3_^2−^ generated by the hydration of cement [40]. The above observations are consistent with the references [41,42]. Although parts of absorption peaks present some slight offset in CEACB comparing with FTIR spectra of EAER and cement powder, this is merely caused by the physical coexistence of cement and emulsified asphalt. The new functional groups appeared in the CEACB spectrum is attributed to the cement hydration. As a result, the infrared spectrum of CEACB resembles a superposition of cement powder and emulsified asphalt, indicating that the cement-emulsified asphalt interaction is a physical blending process.

**Figure 2:**
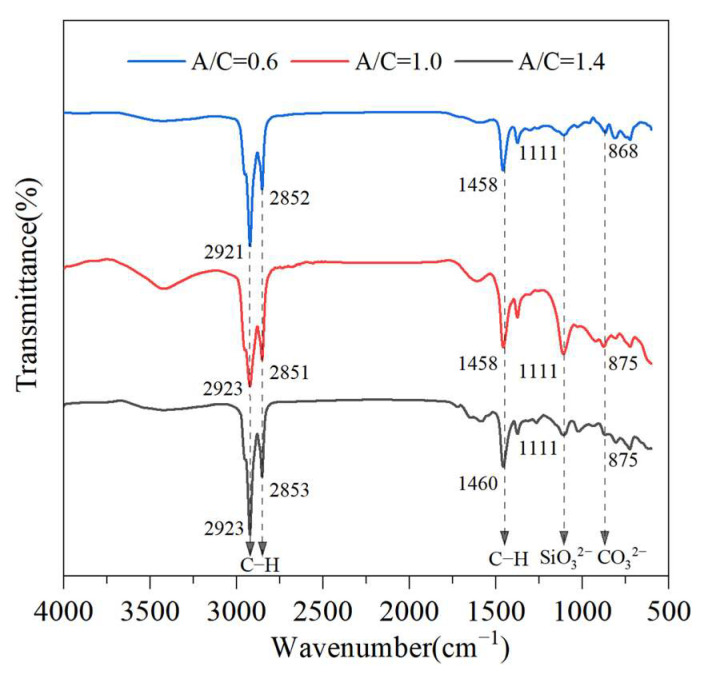
FTIR spectra of CEACB with different A/C ratios.

To further explore the influence of cement proportion on the cement-emulsified asphalt interaction, the characteristic peaks of CEACB with the three kinds of A/C ratios of 0.6, 1.0, and 1.4 were analyzed as shown in Figure 2. It could be seen that the curve of each FTIR spectrum comprises five characteristic peaks. For instance, when A/C = 0.6, the wavenumber of 2921 cm^−1^ and 2852 cm^−1^ denote the stretching vibration absorption peaks of C–H. The bending vibration absorption peak of C–H appears at 1458 cm^−1^. The absorption peak of the SiO_3_^2−^ group is observed at 1111 cm^−1^. The bending vibration absorption peak of CO_3_^2−^ appears at 868 cm^−1^. Similar results were also observed in the infrared spectra at the A/C ratio of 1.0 and 1.4, demonstrating that there is no substantial variation in position, intensity and shape of the functional groups. The results reveal that the physical blending properties of the cement-emulsified asphalt interaction would be regardless of the cement proportion in CEACB. 

### 3.2. Micro-Morphological Characteristics of CEACB 

**Figure 3:**
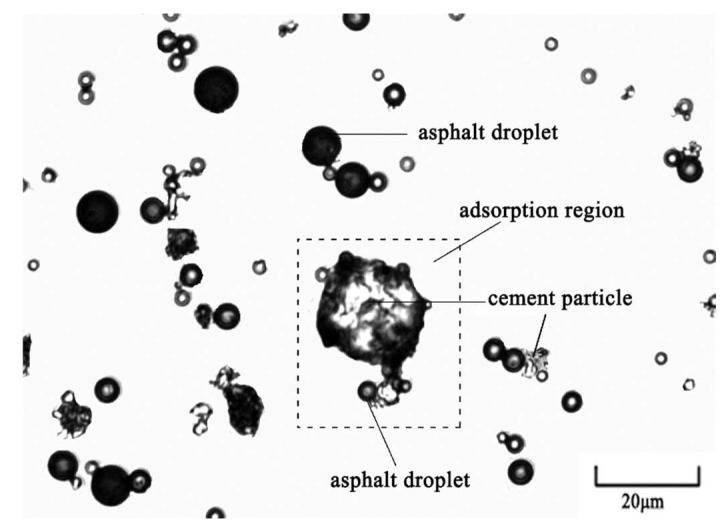
Micro-morphology of particles in fresh CEACB.

The micro-morphological characteristic of fresh CEACB was observed by FM as shown in Figure 3, which exhibits a typical micro-morphological state of early-age CEACB. The results indicate that the distribution of particles including asphalt droplets and cement particles is mainly in a dispersed state as for fresh CEACB. The circle zone is asphalt droplets, and the irregularly shaped zone is cement particles. In addition, the adsorption between cement particles and asphalt droplets and the aggregation among asphalt droplets are also observed in small amounts. The observed micro-morphological characteristics of fresh CEACB present similar results with other traditional cement emulsified asphalt materials as mentioned in previous studies through FM method [43,44]. The formation mechanism of the micro-morphologies during the continuous process of cement hydration and emulsified asphalt demulsification has been employed to reveal the cement-emulsified asphalt interaction of early-age CEACB through the micro-morphological variance characteristics of aggregation region and adsorption region in CEACB.

**Figure 4:**
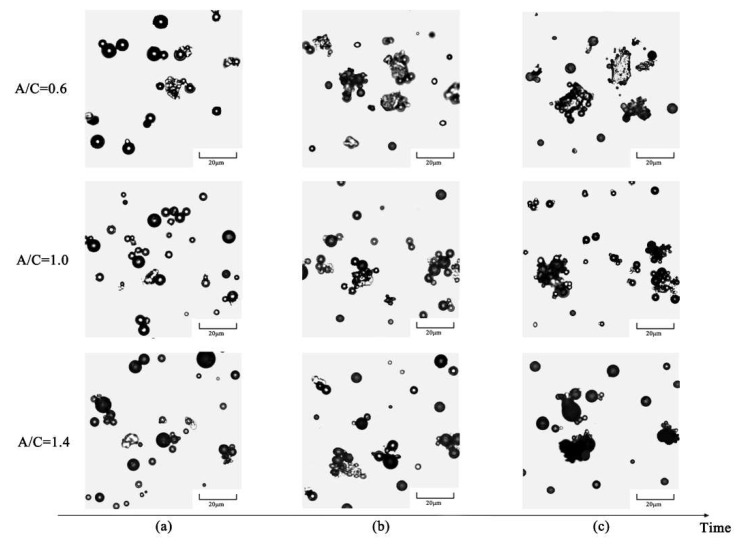
Micro-morphological evolution characteristics of CEACB. (**a**) 0 min, (**b**) 60 min, (**c**) 120 min.

As a high-polar material, cement could exert a stronger adsorption capacity for asphalt droplets in CEACB [45]. The micro-morphological evolution characteristics of asphalt droplets with different A/C ratios are illustrated in Figure 4. The results show from Figure 4a that the morphologies of asphalt droplets and cement particles in the three kinds of fresh CEACB present a relatively even dispersion state, which indicates that the cement-emulsified asphalt interaction is not obvious in the early-age state of CEACB.

At the time of 60 min, cationic asphalt droplets with a positive charge in the three kinds of CEACB begin to orient themselves toward cement particles with a negative charge due to the effect of electrostatic adsorption, and even some asphalt droplets clustered together are adsorbed on the surface of cement particles, gathering into an irregular adsorption region as presented in Figure 4b. Meanwhile, it is observed that the A/C ratio could exert a significant influence on micro-morphological characteristics of CEACB at this time, which is mainly determined by the hydration of cement particles and the adsorption of cement particles to asphalt droplets. When A/C = 0.6, the irregular adsorption region of cement particles to asphalt droplets exhibits a trend of rapid expansion due to cement hydration compared with the fresh state of CEACB. Each cement particle would adsorb small quantities of asphalt droplets with small particle size due to the competitive adsorption effect between cement particles presenting a state of being locally covered by asphalt droplets. When A/C = 1.4, many asphalt droplets are absorbed on the surface of cement particles, exhibiting a state of complete coverage, which would induce cement particles to be difficult to contact with water and reduce the hydration degree of cement particles. The irregular adsorption region also becomes larger but is close to or less than that of the A/C ratio of 0.6. At the A/C ratio of 1.0, the adsorption effects of cement particles to asphalt droplets and the expansion degree of the irregular adsorption region fall between the above two A/C ratios. It is believed that this interaction behaviors of cement-emulsified asphalt would provide a favorable expectation for the continuous hydration of cement particles and the complete demulsification of asphalt droplets.

Over time, the adsorption region of cement particles to asphalt droplets presents a darker gray level and gradually evolves into a flocculation state at the time of 120 min as illustrated in Figure 4c, which indicates the demulsification occurrence of asphalt droplets due to the diffusion of trapped water from asphalt droplets. However, the micro-morphological images have great differences among the three kinds of CEACB. When A/C = 0.6, it is observed that cement particles are partially covered with thinner asphalt film and the boundary of adsorption region is dominated by edges and corners of cement particles and is locally attached with asphalt droplets with small size. The irregular adsorption region has a slight increase attributed to the sufficient demulsification of small quantities of asphalt droplets adsorbed on the surface of cement particles and the slow hydration of cement particles from outside to inside. Due to more asphalt droplets existing in CEACB with the A/C ratio of 1.4, the adsorption of cement particles to asphalt droplets achieves a certain saturation state. Cement particles are completely encapsulated by thicker asphalt films and excessive coalescent asphalt droplets. The adsorption region of cement particles to asphalt droplets has a relatively smooth boundary. Compared with the A/C ratio of 0.6, the slight increase in the irregular adsorption region is due to the further aggregation of asphalt droplets toward the adsorption region and the demulsification of large amounts of asphalt droplets adsorbed on the surface of inadequately hydrated cement particles. When A/C = 1.0, the size of the irregular adsorption region presents the most rapid increase than that of the other two A/C ratios. The boundary of adsorption region presents the characteristic of continuous smooth envelope line and edges and corners of cement particles. The main reason for this observation attributes to the sufficient demulsification of asphalt droplets and the stable hydration of cement particles from outside to inside, which implies that this interaction ability of cement-emulsified asphalt achieves the optimal state.

**Figure 5:**
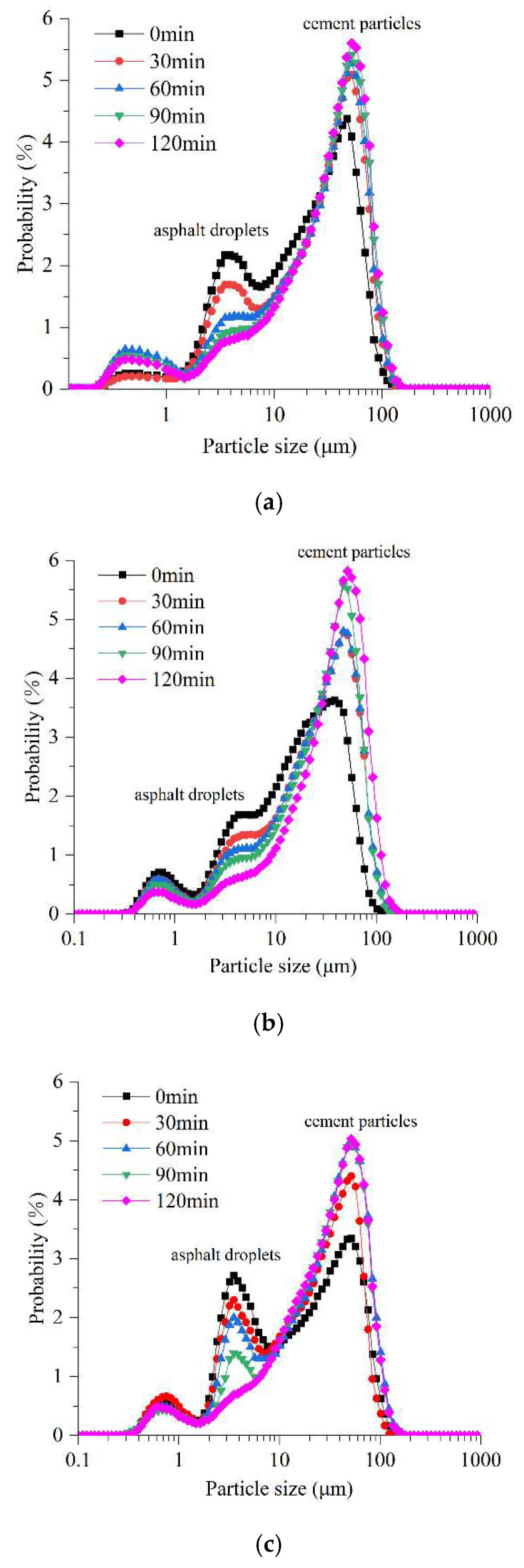
Distribution characteristics of cement particles and asphalt droplets in CEACB. (**a**) A/C = 0.6, (**b**) A/C = 1.0, (**c**) A/C = 1.4.

To quantitatively investigate interaction behaviors of cement-emulsified asphalt of early-age CEACB, the characteristics of particle size distribution (PSD) coupling with the micro-morphological evolution of asphalt droplets were explored with the employment of LPSA. As shown in Figure 5, the PSD characteristics of three kinds of CEACB are similar to the results of previous studies [34]. It can be also seen from Figure 5 that the peak values of asphalt droplets and cement particles are around 3–5 μm and 20–30 μm, respectively. As far as all CEACB is concerned, the peak of asphalt droplets gradually decreases and eventually tends to disappear, and the peak of cement particles presents a stable rising trend within the time of 0–120 min, which indicates that the PSD characteristics of all CEACB exhibit the conversion from poor stability suspension system to good stability suspension system [46]. Besides, the peak of cement particles of all CEACB would shift to the right to some extent. By analysis, the observed phenomenon could be attributed to the volume increase in cement particles induced by intensive cement hydration, the adsorption of cement particles with high polarity to asphalt droplets with small size, and the demulsification of asphalt droplets resulting from failure of double layer structure of asphalt droplets [32,33]. However, it is observed that the A/C ratio could have a significant impact on the PSD of CEACB characterized by the probability change of the size of asphalt droplets and cement particles, which can be employed to indirectly reflect the interaction behaviors of cement-emulsified asphalt of early-age CEACB.

Comparing the other two A/C ratios, the peak of asphalt droplets has the most prominent decline and the peak of cement particles presents a considerable upward trend and shifts slightly to the right at the A/C ratio of 0.6 within the time of 0–60 min. The observation can be concluded that the dramatic adsorption ability of cement particles to asphalt droplets under the condition of the high cement proportion would induce the decline of number of suspended asphalt droplets. Meanwhile, the formed adsorption region between cement particles and asphalt droplets and the volume expansion of cement particles owing to cement hydration would accelerate the variation of the peak of cement particles. With a time increase from 60 min to 120 min, the peaks of cement particles and asphalt droplets exhibit slight differences due to the formation of thin asphalt films generating from the demulsification of the small amounts of asphalt droplets adsorbed on the surface of cement particles, providing less contribution to the expansion of the absorption region. 

When A/C = 1.4, the peak of asphalt droplets shows a slight downward trend within the time of 0–60 min since only parts of abundant asphalt droplets dispersed in CEACB are adsorbed by a small amount of cement particles and the saturation adsorption level of cement particles to asphalt droplets is rapidly reached. Hereafter, it is observed that the peak of asphalt droplets rapidly drops due to the conversion of adsorption behavior from the adsorption between cement particles and asphalt droplets to the coalescence among asphalts droplets. The peak of cement particles exhibits the increasing trend in terms of the expansion of the adsorption zone along with the hydration of cement and the coalescence of asphalt droplets orienting to the cement hydration products. However, comparing with the A/C ratio of 0.6, the peak of cement particles shifts to the right slightly in that excessive asphalt droplets would encapsulate the surface of cement particles with thicker asphalt film to hinder the connection of water and cement particles and weaken the degree of cement hydration and asphalt droplet demulsification. 

When A/C = 1.0, the peaks of asphalt droplets and cement particles present similar change characteristic as the other two A/C ratios throughout the observation period. Noteworthily, there exists the widest offset to a larger size direction as for the peak of cement particles from initial state to eventual state, demonstrating the desired interaction of cement-emulsified asphalt of early-age CEACB to promote the formation of the hydration of cement particles from outside to inside, the adsorption of cement particles to asphalt droplets, and the demulsification of asphalt droplets. By analysis, it is believed that the combination of the two testing techniques is a valuable perspective to explore micro-morphological evolution characteristics, interpret the interaction mechanism of cement-emulsified asphalt of early-age CEACB with different A/C ratios, and establish an auxiliary decision-making method for the preparation of CEACB. 

### 3.3. Microstructural Properties of CEACB 

**Figure 6:**
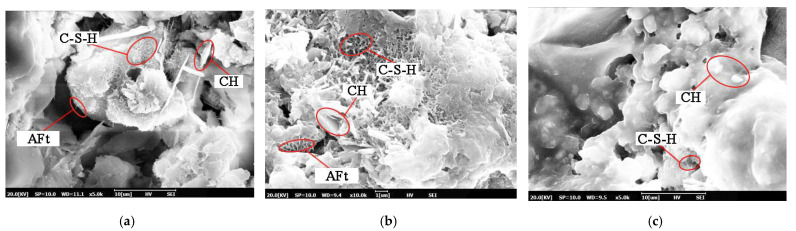
Microstructural characteristics of CEACB at curing time of 1 day. (**a**) A/C = 0.6, (**b**) A/C = 1.0, (**c**) A/C = 1.4.

The microstructural characteristics of CEACB have attracted great attention for revealing the process of the strength development through SEM with the extension of curing time, which is closely related to the spatial network structure of CEACB along with cement hydration products growing and interlacing with asphalt film. As shown in Figure 6, the hydration products such as ettringite (AFt), calcium silicate hydrates (C-S-H) gel and calcium hydroxide (CH) and so on could be clearly observed after curing time of 1 day, which implies the formation of the initial cement hydration process. 

When A/C = 0.6, the morphology of loose and porous texture with large voids is presented in the microstructural images of CEACB, which results from the intense agglomeration effect of cement and hydration products and the lack of asphalt films to wrap all the hydration products and cement. This microstructural state demonstrates that the relatively high cement proportion would weaken the interaction ability of the cement-emulsified asphalt. When A/C = 1.4, it is observed that the microstructure exhibits the characteristics with protuberant surface and local large voids, and cement particles are fully wrapped by thicker and continuous asphalt films, which would delay the formation process of hydration products due to the inhibition of moisture transfer. Comparing with A/C ratios of 0.6 and 1.4, the microstructure tends to form a complex spatial network structure along with hydration products, such as needle-like AFt and cluster C-S-H, growing and interweaving with asphalt films at the A/C ratio of 1.0.

**Figure 7:**
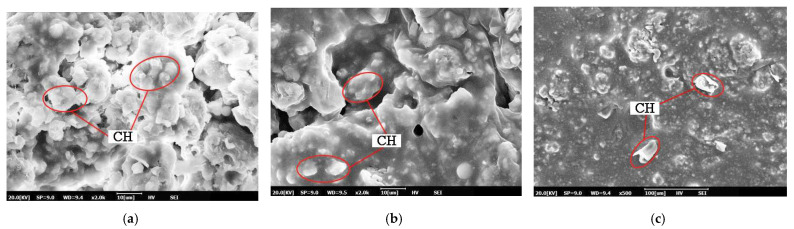
Microstructural characteristics of CEACB at curing time of 7 days. (**a**) A/C = 0.6, (**b**) A/C = 1.0, (**c**) A/C = 1.4.

Meanwhile, the microstructural characteristics of CEACB with different A/C ratios are also scanned after a curing time of 7 days to investigate the interaction behaviors of cement-emulsified asphalt during the process of strength development. It can be seen from Figure 7 that the microstructural form of CEACB becomes denser, attributed to the ever-growing hydration products and the sufficient demulsification of emulsified asphalt with the extension of curing time. 

The main reason for this fact is that the ever-growing hydration products gradually lap together and build the skeleton of CEACB along with the decreased production in needled Aft and increase in sheet CH. Additionally, the continuous cement hydration and moisture evaporation process accelerate the formation of more asphalt films, which are adequately interweaved with hydration products, playing a dominant role in wrapping hydration products and a small amount of unhydrated cement particles. The two aspects including the reinforcing effect of hydration products and the bonding behavior of asphalt films could further enhance the dense state of CEACB, indirectly reflecting the improvement of interaction ability of cement-emulsified asphalt. The observation results could be fully illustrated when A/C = 1.0. 

However, when A/C = 0.6, the stacking phenomenon of cement and hydration products becomes more obvious and the local voids tend to be smaller than the curing time of 1 day, but larger than the other two A/C ratios at curing time of 7 days. When A/C = 1.4, the microstructure surface presents a smoother state and large amount of asphalt films excessively wrap cement particles and hydration products and partial free asphalt floats outside the skeleton structure. Undoubtedly, the reasonable A/C ratio would exert great influence on the formation of denser microstructural morphology and the improvement of the interaction between cement and emulsified asphalt, which is beneficial to the strength development of CEACB. 

### 3.4. Evaluation of Cement-Emulsified Asphalt Interaction

#### 3.4.1. Evaluation Method

Generally, CEACB can be considered as a typical multi-phase composite material, in which emulsified asphalt is a continuous phase and cement is a dispersed phase. The macro-rheological properties of CEACB are significantly affected by the cement-emulsified asphalt interaction, which would exhibit different characteristics due to the different structural composition of CEACB. Consequently, establishing a quantitative method based on the rheological properties of CEACB to evaluate the interaction ability of cement-emulsified asphalt would have the potential benefits to improve the mechanical performance of CEACB. As mentioned in previous researches, a serial of rheological indexes, such as K-B-G*, *L-A-δ* and *K-B-δ*, etc., deriving from tests of DSR have been also extensively utilized to explore the interaction ability of asphalt-mineral filler for hot mix asphalt [47,48,49,50]. It is worth mentioning that the *K-B-*$G^{*}$ index proposed by *K*. Ziegel and A. Romanov [51] as shown in Equation (1) has been proven to be a more appropriate index to analyze the interaction ability of continuous phase and dispersed phase than other indexes. The greater value of *K-B-*$G^{*}$ is, the stronger interaction ability of cement-emulsified asphalt is.

(1) $$K\text{-}B\text{-}G^{*}=\frac{(G_{c}^{*}/G_{m}^{*})-1}{(1.5+G_{c}^{*}/G_{m}^{*})\cdot \varphi_{f}}$$

where, $G_{c}^{*}$ is the complex modulus of CEACB; $G_{m}^{*}$ is the complex modulus of emulsified asphalt; $\varphi_{f}$ is the total volume fraction of cement hydration products and unhyrated cement particles with a characteristic of dynamic change along with the hydration of cement.

As presented in results of the above FTIR experiment, only cement particles in CEACB undergo the chemical reaction of the hydration process and the demulsification of emulsified asphalt is only a physical change. Assumed that the volume fraction of asphalt phase keeps constant during the process of demulsification of emulsified asphalt, the value of $\varphi_{f}$ has a close relation with the volume fraction of cement paste (cement particles and water) in fresh CEACB and can be obtained as follows [52,53]: 

The volume fraction of cement paste ($V_{CP}$) in fresh CEACB can be expressed as Equation (2).

(2) $$V_{CP}=\frac{m_{C}/\rho_{C}+m_{W}/\rho_{W}}{m_{C}/\rho_{C}+m_{W}/\rho_{W}+m_{A}/\rho_{A}}$$

where, $m_{C}$ is the mass of cement filler; $m_{A}$ is the solid content of emulsified asphalt; $m_{W}$ is the mass of mixing water; $\rho_{C}$ is the density of cement filler and is about equal to 3.080 g/cm^3^; $\rho_{A}$ is the density of asphalt and equals approximately to 1.030 g/cm^3^; $\rho_{W}$ is the density of mixing water and equals to 1.000 g/cm^3^.

The volume fraction of unhydrated cement particles ($V_{CS}$) in CEACB is listed as Equation (3).

(3) $$V_{CS}=V_{CP}\cdot (1-p)\cdot (1-\alpha )$$

where, p is the volume fraction of mixing water obtained from Equation (4); α is hydration degree of cement as presented in Equation (5) and can be tested in accordance with the reference [53]. In the study, the mean value of three replicate samples in each case was employed to characterize the actual degree of cement hydration.

(4) $$p=\frac{m_{W}/\rho_{W}}{m_{C}/\rho_{C}+m_{W}/\rho_{W}}$$

(5) $$\alpha =\frac{m_{n}}{m_{C}}=\frac{m_{2}-m_{1}}{0.23\cdot m_{2}}$$

where, $m_{n}$ is the mass of the chemically combined water in cement hydration products; $m_{1}$ and $m_{2}$ are masses of samples before and after being calcined, respectively; and 0.23 is the theoretical value of chemical combined water in per unit cement hydration products. 

The volume fraction of cement hydration products ($V_{GS}$) in CEACB is calculated as Equation (6).

(6) $$V_{GS}=1.52\cdot (1-p)\cdot \alpha \cdot V_{CP}$$

The volume fraction of C-S-H gel water of cement hydration products ($V_{CW}$) is expressed as Equation (7).

(7) $$V_{CW}=0.60\cdot (1-p)\cdot \alpha \cdot V_{CP}$$

Gel water with the larger bonding energy is invariably adsorbed on the surface of C-S-H gel pores and its desorption would occur only above 105 °C; thus, C-S-H gel water can be considered as one part of cement hydration products. Therefore, the total volume fraction of cement hydration products ($V_{CH}$) can be obtained from Equation (8).

(8) $$V_{CH}=2.12\cdot (1-p)\cdot \alpha \cdot V_{CP}$$

Combining Equations (3) and (8), the total volume fraction of cement hydration products and unhydrated cement particles ($\varphi_{f}$) is described as Equation (9).

(9) $$\varphi_{f}=V_{CS}+V_{CH}=V_{CP}\cdot (1-p)\cdot (1+1.12\alpha )$$

#### 3.4.2. Evaluation Results

**Figure 8:**
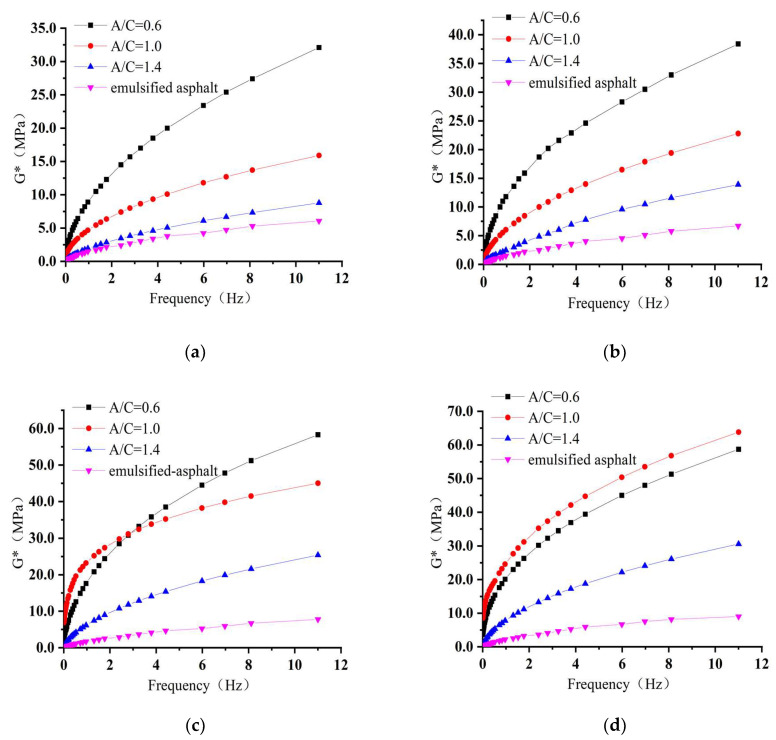
Frequency sweep curves for emulsified asphalt and CEACB with different curing time. (**a**) Curing time of 1 day, (**b**) Curing time of 3 days, (**c**) Curing time of 7 days, (**d**) Curing time of 14 days.

Figure 8 illustrates the complex shear modulus (G*) of CEACB and emulsified asphalt with different A/C ratios and curing time through frequency sweep tests of DSR at a control temperature of 25 °C. As shown in Figure 8, the value of G* of each CEACB increases with increasing of the loading frequency regardless of cement proportion or curing time. When the A/C ratio remains the same, the G* of CEACB increases gradually with the extension of curing time. Specifically, in Figure 8a,b, the growth rate of G* of CEACB with a A/C ratio of 0.6 is higher than those of CEACB with A/C ratios of 1.0 and 1.4 at curing time of 1 day and 3 days. However, the values of G* of CEACB with an A/C ratio of 1.0 are greater within the low frequency range after curing time of 7 days as shown in Figure 8c. Moreover, as observed from Figure 8d, the values of G* under a 1.0 A/C ratio are the largest among three A/C ratios. It is worth mentioning that the increase of G* values of CEACB with A/C ratio of 1.4 is relatively gentle and always lower than the other two groups. On the basis of results of G*, it demonstrates that an appropriate A/C ratio in the full of curing time plays a significant role in dominating the viscoelastic behaviors of CEACB. 

**Figure 9:**
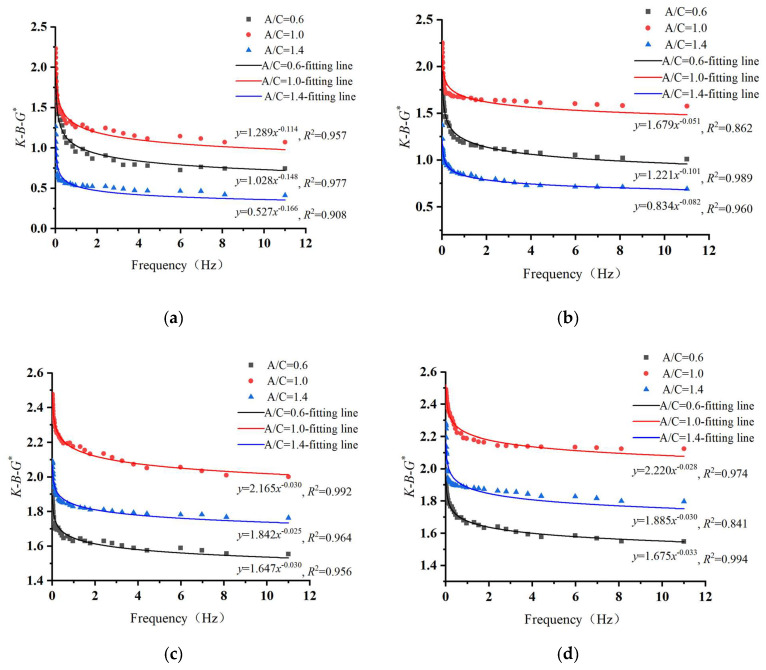
*K-B-*$G^{*}$ of CEACB with different A/C ratios and curing time. (**a**) Curing time of 1 day, (**b**) Curing time of 3 days, (**c**) Curing time of 7 days, (**d**) Curing time of 14 days.

The $\varphi_{f}$ values of CEACB with different A/C ratios and curing time are presented in Table 3 according to the Equations (4), (5) and (8), which were input into the Equation (1) together with the above results of G* of CEACB and emulsified asphalt. Consequently, the curves of *K-B-*$G^{*}$ of CEACB under the condition of different A/C ratios and curing time over the full frequency range can be calculated as shown in Figure 9. The relationship between *K-B-*$G^{*}$ and loading frequencies was established by fitting with power function. The pre-exponential factor of power function was utilized to reflect the effects of A/C ratio, and curing time on the cement-emulsified asphalt interaction and the greater pre-exponential factor is close to the stronger interaction ability [54]. 

The results show that the values of *K-B-*$G^{*}$ of three kinds of CEACB are all susceptible to loading frequencies and present a decreasing trend with the increase in loading frequency. This is consistent with the research results of Liu [49], in which the interaction ability between asphalt and filler had been proved to have the similar variation as loading frequency increases from 1 Hz to 100 Hz through the utilization of the *K-B-*$G^{*}$ index. The pre-exponential factors of the fitting curves of *K-B-*$G^{*}$ values of CEACB with A/C ratio of 1.0 are 1.289, 1.679, 2.165 and 2.220 after the curing time of 1 day, 3 days, 7 days and 14 days, respectively, presenting the highest values among the three kinds of CEACB, which demonstrates that the ability of cement-emulsified asphalt interaction of CEACB with A/C ratio of 1.0 is the strongest. This result reflects that the hydration reaction and the demulsification process of CEACB are more sufficient at A/C ratio of 1.0, which is consistent with the above SEM results. 

In addition, under the condition of the same A/C ratio, the values of *K-B-*$G^{*}$ of CEACB significantly increase with the extension of curing time from 1 day to 7 days and then basically remain stable after 7 days. The pre-exponential factors of the fitting curves of *K-B-*$G^{*}$ values of CEACB with A/C ratio of 0.6, 1.0 and 1.4 have a lift from 1.028 to 1.675, 1.289 to 2.220 and 0.527 to 1.885 with the increase of curing time from 1 day to 14 days, respectively. The main reason for this fact is that an adequate curing time promotes the interaction ability of cement and emulsified asphalt, leading to a denser spatial network structure of CEACB. Therefore, the cement proportion greatly affects the interaction behaviors of cement-emulsified asphalt and the *K-B-*$G^{*}$ could be utilized to evaluate the interaction ability of cement-emulsified asphalt. Selecting a reasonable A/C ratio is very crucial to improve the mechanical performance of CEACB as presented in the results of the mechanism of cement-emulsified asphalt interaction associated with microstructural characteristics of CEACB.

## 4. Conclusions

The interaction mechanism and behaviors of cement-emulsified asphalt from the whole process of fresh state to strength formation were investigated systematically from a multi-scale perspective of microstructural morphology evolution process and macro-rheological properties of CEACB. The main conclusions are presented as follows.

(1) FTIR spectra could be utilized as an effective approach for reflecting the essence of physico-chemical processes of cement-emulsified asphalt interaction. No new substances observed in the CEACB spectrum regardless of the cement hydration products indicates that the cement-emulsified asphalt interaction is a physical blending process.

(2) The interaction behaviors of cement-emulsified asphalt of early-age CEACB could be interpreted by the characteristics of micro-morphological evolution and the variation of particle size distribution. The influence of cement content on the initial behaviors of the cement hydration and the emulsified asphalt demulsification could be characterized by the aggregation process among asphalt droplets and the adsorption behaviors of cement particles to asphalt droplets. 

(3) The microstructural characteristic of CEACB along with cement hydration products growing and interlacing with asphalt films with the extension of curing time could be observed by the SEM. The appropriate cement proportion would significantly enhance the formation of the dense spatial network structure to promote the strength improvement of CEACB.

(4) The macro-rheological index *K-B-*$G^{*}$ with full consideration of cement hydration process is very appropriate for evaluating the interaction ability of cement-emulsified asphalt under the condition of different cement proportions and curing time, the results of which highly correlates with the tests of FM and SEM.

## Figures and Tables

**Table 1:** Chemical composition of cement.

Composition	Weight Percentage
CaO	61.13
SiO_2_	24.48
Al_2_O_3_	5.05
MgO	2.33
SO_3_	2.04
Fe_2_O_3_	3.38
K_2_O_3_	0.54
Na_2_O	0.77
TiO_2_	0.08

**Table 2:** Properties of emulsified asphalt.

Properties	Unit	Value	Test Method
Test on Emulsified Asphalt			
Sieve test (1.18 mm)	%	0.01	ASTM D244
Storage stability (1 day, 25 °C)	%	0.4	ASTM D244
Storage stability (5 days, 25 °C)	%	3.2	ASTM D244
Test on Asphalt Residue			
Solid content	%	60.0	ASTM D244
Penetration (25 °C, 100 g, 5 s)	0.1 mm	75.3	ASTM D5
Ductility (25 °C)	cm	80.4	ASTM D113
Kinematic viscosity	Pa∙s	95.2	ASTM D2170
Softening point	°C	49.5	ASTM D36

**Table 3:** Volume fraction values of cement hydration products and unhydrated cement particles.

A/C Ratio	Curing Time (Day)	*P*	α		$\varphi_{f}\;(\%)$
			Mean	Std. Dev.	
0.6	1	0.55	0.30	0.02	33.30
	3	0.55	0.52	0.03	39.36
	7	0.55	0.70	0.04	44.27
	14	0.55	0.71	0.02	44.69
1.0	1	0.67	0.26	0.01	21.43
	3	0.67	0.49	0.02	25.61
	7	0.67	0.67	0.04	28.87
	14	0.67	0.68	0.04	29.18
1.4	1	0.74	0.19	0.02	15.10
	3	0.74	0.37	0.01	17.57
	7	0.74	0.56	0.04	20.21
	14	0.74	0.58	0.02	20.46
